# Performance of Unobserved Self-Collected Nasal Swabs for Detection of SARS-CoV-2 by RT-PCR Utilizing a Remote Specimen Collection Strategy

**DOI:** 10.1093/ofid/ofab039

**Published:** 2021-01-28

**Authors:** Ron M Kagan, Amy A Rogers, Gwynngelle A Borillo, Nigel J Clarke, Elizabeth M Marlowe

**Affiliations:** 1 Quest Diagnostics Infectious Disease, San Juan Capistrano, California, USA; 2 Quest Diagnostics Nichols Institute, San Juan Capistrano, California, USA

**Keywords:** SARS-CoV-2, COVID-19, self-collection, RT-PCR, cycle threshold

## Abstract

**Background:**

The use of a remote specimen collection strategy employing a kit designed for unobserved self-collection for severe acute respiratory syndrome coronavirus 2 (SARS-CoV-2) reverse transcription polymerase chain reaction (RT-PCR) can decrease the use of personal protective equipment (PPE) and exposure risk. To assess the impact of unobserved specimen self-collection on test performance, we examined results from a SARS-CoV-2 qualitative RT-PCR test for self-collected specimens from participants in a return-to-work screening program and assessed the impact of a pooled testing strategy in this cohort.

**Methods:**

Self-collected anterior nasal swabs from employee return-to-work programs were tested using the Quest Diagnostics Emergency Use Authorization SARS-CoV-2 RT-PCR. The cycle threshold (Ct) values for the N1 and N3 N-gene targets and a human RNase P (RP) gene control target were tabulated. For comparison, we utilized Ct values from a cohort of health care provider–collected specimens from patients with and without coronavirus disease 2019 symptoms.

**Results:**

Among 47_ _923 participants, 1.8% were positive. RP failed to amplify for 13/115_ _435 (0.011%) specimens. The median (interquartile range) Cts were 32.7 (25.0–35.7) for N1 and 31.3 (23.8–34.2) for N3. Median Ct values in the self-collected cohort were significantly higher than those of symptomatic but not asymptomatic patients. Based on Ct values, pooled testing with 4 specimens would have yielded inconclusive results in 67/1268 (5.2%) specimens but only a single false-negative result.

**Conclusions:**

Unobserved self-collection of nasal swabs provides adequate sampling for SARS-CoV-2 RT-PCR testing. These findings alleviate concerns of increased false negatives in this context. Specimen pooling could be used for this population, as the likelihood of false-negative results is very low when using a sensitive, dual-target methodology.

The ongoing coronavirus disease 2019 (COVID-19) pandemic has placed unpredicted strains on the US health care system, resulting in an extraordinary worldwide demand for laboratory testing. Nucleic acid amplification testing (NAAT) for severe acute respiratory syndrome coronavirus 2 (SARS-CoV-2) from upper respiratory swabs is a critical tool for addressing the pandemic. The current US Centers for Disease Control and Prevention (CDC) Interim Guidelines for Collecting, Handling, and Testing Clinical Specimens for COVID-19 (https://www.cdc.gov/coronavirus/2019-ncov/lab/guidelines-clinical-specimens.html) include nasopharyngeal (NP) or oropharyngeal (OP) swabs collected by a health care provider (HCP) or nasal (midturbinate, anterior nares) swabs collected by an HCP or self-collected (SC) by the patient either unsupervised at home or supervised on-site. Recent studies have shown that patient self-collected nasal swabs are comparable to HCP-collected swabs for SARS-CoV-2 testing [1–4]. Self-collection of swab samples for SARS-CoV-2 testing may be beneficial to limit the use of personal protective equipment (PPE), reduce the risk of infection to health care workers and other patients, and offer additional opportunities to provide services and decrease the burden on clinics. In addition, SARS-CoV-2 testing by reverse transcription polymerase chain reaction (RT-PCR) utilizing unobserved self-collected nasal swabs is an important return-to-work tactic to help ensure a safe work environment, supporting the nation’s economic viability. As employees transition from a working-from-home model, they may undergo voluntary screening before returning to work. This demographic represents a low–disease prevalence population, and pooled RT-PCR testing could afford a more efficient and rapid way to perform screening. In a previous study, we demonstrated that pooled and individual testing of specimens positive for SARS-CoV-2 demonstrated 100% agreement [5]. Quantitative comparisons between RT-PCR cycle threshold (Ct) values of internal adequacy controls have been used to evaluate differences in adequacy between HCP-collected and self-collected respiratory samples in a number of studies [6–8]. In the current study, we examined the Ct value distribution of an Rnase P (RP) specimen adequacy control to further improve our understanding of the ability of patients to self-collect specimens utilizing an alternate delivery strategy. In addition, we examined the distribution of Ct values among unobserved self-collected nasal swabs to assess whether using pooled, rather than singlicate, testing would have increased the risk of false-negative test results.

## METHODS

In this retrospective study, we tabulated the Ct values for a cohort of unobserved self-collected nasal swabs tested in singlicate between June and August 2020 using the Quest Diagnostics SARS-CoV-2 qualitative RT-PCR authorized for emergency use (EUA) by the US Food and Drug Administration (FDA) for self-collection. This test utilizes a 1-step reverse transcription and PCR amplification with SARS-CoV-2-specific primers and real-time detection with SARS-CoV-2-specific probes for the N1 and N3 targets of the virus nucleocapsid gene. This test utlizes an in-house prepared exogenous N-gene transcript that serves as an internal extraction and amplification control. For these unobserved self-collected specimens, an independent human RNAse P (RP) gene target to verify adequacy of nasal swab self-collection was included in the assay in a separate well (https://www.fda.gov/media/136231/download). Nasal swab self-collection was performed using an FDA EUA self-collection kit. Briefly, collection kits were mailed to participants with instructions to collect bilateral nasal swabs and sent back to the testing laboratory via FedEx as described in the instructions for use (https://www.fda.gov/media/138402/download).

HCP-collected specimens were submitted to Quest Diagnostics from providers primarily in the United States including from hospitals, health maintenance organizations, and private practitioners. N1 and N3 Ct values for all SARS-CoV-2-positive HCP-collected specimens tested during the same period and selected from patients spanning the same range of ages as for the self-collected specimens were tabulated for comparison. Additionally, we tabulated Ct values for HCP-collected specimens that included an optional indication of whether the patient was symptomatic for COVID-19. These specimens were tested between March 24 and May 13, 2020, and selected from patients spanning the same range of ages as for the self-collected specimens. HCP-collected specimen types included nasopharyngeal, oropharyngeal, and nasal swabs as well as bronchoalveolar lavage, but were not recorded at the time of the test order submission.

A specimen was deemed positive for SARS-CoV-2 RNA if the Ct values for both targets were <40 cycles, negative if the Ct values for both targets were ≥40 cycles and the internal amplification control was valid, and inconclusive if the Ct value was <40 cycles for only 1 detector. Specimens with inconclusive results were retested in singlicate from the original swab. In self-collected specimens that were negative for the SARS-CoV-2 N1 and N3 targets, the test was deemed invalid if the RP amplification was not evident (Ct value ≥40) in the initial test run and upon repeat. Patient age, gender, and Ct values for N1, N3, and RP were tabulated in a database, and the distributions of Ct values were evaluated and compared with the shift in the Ct value cutoff determined for pooled testing (40 cycles—Ctshift) using the Quest SAR-CoV-2 RT-PCR EUA assay [5].

Statistical analysis was performed using Analyze-it for Microsoft Excel, version 5.65.9. Proportions were compared with the Pearson chi-square test. Continuous variables were compared with the Kruskal-Wallis test or the Wilcoxon-Mann-Whitney test. The Hodges-Lehmann estimator was used to estimate median Ct value shifts and 95% confidence intervals.

### Patient Consent Statement

This study utilized retrospectively collected, de-identified data from previously tested specimens. No human subjects were utilized in this study, and thus patient consent was not applicable.

## RESULTS

We tabulated SARS-CoV-2 RT-PCR results from 115_ _435 specimens obtained by unsupervised self-collection from 47_ _923 participants (men: 70.6%) reported between June 3 and August 12, 2020. Fifty percent of the participants in this cohort submitted a single specimen, 12.3% submitted 2 specimens, 10.5% submitted 3 specimens, and 27.3% submitted ≥4 specimens (maximum, 9). The median Ct value for the RP sampling adequacy target was 23.0 for the positive and 23.4 for the negative specimens, and the 99.5th percentile for RP Ct values was <30, indicating adequate nasal swab sampling for the vast majority of specimens (Table 1). The 99.5th percentile of the RP Ct values for 110 specimens with an inconclusive report was also <30, indicating adequate sampling independent of an inconclusive SARS-CoV-2 test result. The coefficient of variation (CV) of RP Ct values for 18_ _083 patients with ≥3 test results was 5.2% ± 2.9% (SD), indicating relative uniformity of intrapatient specimen self-collection. Invalid results were obtained for 13 specimens (0.011%) from 13 participants; these specimens did not show RP amplification despite positive amplification of the specimen internal RT-PCR control. Subsequent testing was performed for 8/13 participants and yielded negative SARS-CoV-2 results. There were no significant differences in RP Ct values between SARS-CoV-2-positive and -negative specimens (data not shown).

**Table 1.** RNase P Ct Value Distributions for Self-Collected SARS-CoV-2-Positive, -Negative, and Inconclusive Specimens

Group^a^	No.	Median Ct (IQR)	99.5th Percentile of RP Ct Values
Positive	1268	23.0 (21.8–24.1)	29.4
Negative	114 057	23.4 (22.2–24.7)	29.8
Inconclusive	110	23.1 (21.9–24.3)	27.6

Abbreviations: Ct, cycle threshold; IQR, interquartile range; RP, RNase P; SARS-CoV-2, severe acute respiratory syndrome coronavirus 2.

^a^See the “Methods” section for the criteria used to classify test results as positive, negative, or inconclusive.

**Figure 1.**
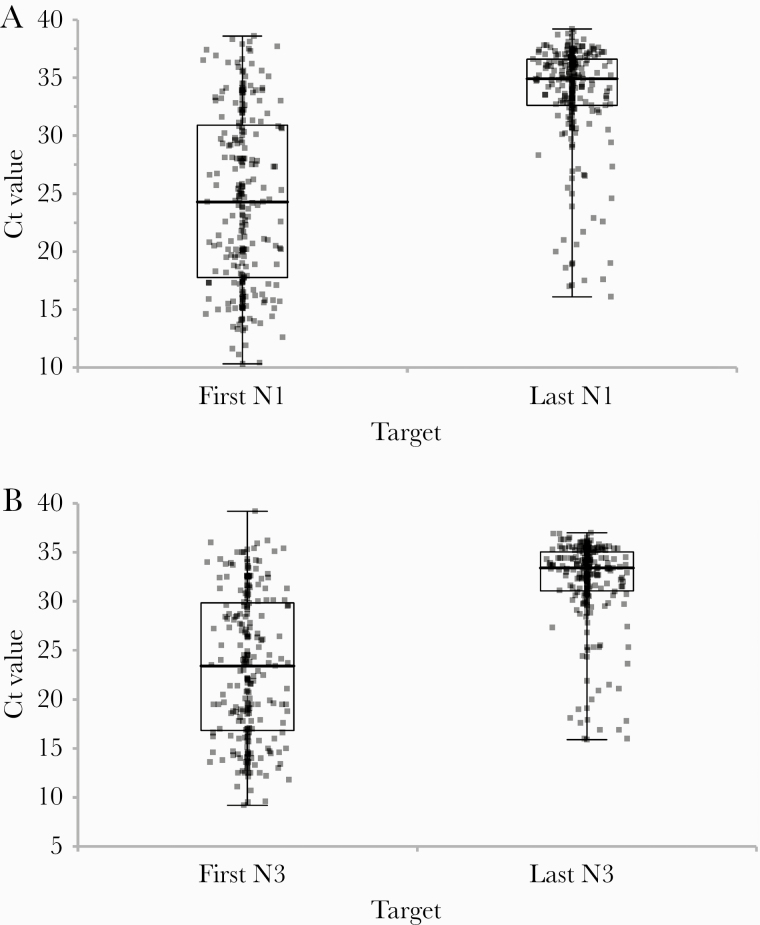
Ct value distributions for the N1 target (A) and N3 target for patients (n = 234) with 2 or more positive SARS-CoV-2 RT-PCR test results. First N1, N3: Ct values from the first positive test. Last N1, N3: paired Ct values for the last positive test. The differences between the first and last Ct value distributions were significant (Wilcoxon, *P* < .0001). Abbreviations: Ct, cycle threshold; IQR, interquartile range; RT-PCR, reverse transcription polymerase chain reaction; SARS-CoV-2, severe acute respiratory syndrome coronavirus 2.

The overall SARS-CoV-2 positivity rate (95% CI) for this cohort was 1.8% (1.7%–1.9%). The positivity rate was higher for women than for men (*P* < .0001) (Table 2). Twenty-seven percent (234/859) of the SARS-CoV-2-positive patients had 2 or more SARS-CoV-2-positive results. The median (IQR) number of days between the first and the last positive result was 11 (7–15) with a maximum of 36 days for all but a single outlier tested again 110 days after the first test. We excluded 6 patients who were tested twice on the same day or only 1 day apart between the first and the last test. The Hodges-Lehmann estimators for the median (95% CI) increase in the Ct values between the first and last positive test were 9.4 (8.3–10.4) for the N1 target and 9.0 (7.9–10.1) for N3 (Figure 1). Overall, the median Ct values for the SARS-CoV-2 nucleocapsid gene targets N1 and N3 were 32.7 and 31.3, respectively, for 1268 positive specimens (Table 3) and did not vary significantly by patient age or by gender (Supplemental Figures 1 and 2). Median RP Ct values showed small variations by age group, ranging from 22.6 to 23.3 (*P* = .016) (Supplementary Figure 3), as well as by gender, with median Ct values of 23.2 for women and 22.8 for men (Hodges-Lehmann shift, 0.5 [0.3–0.7] Cts; *P* < .0001) (Supplementary Figure 4).

**Table 2.** Participant Demographics and SARS-CoV-2 Positivity Rates for Self-Collected Specimens

	Participants^a^			Age, Median (IQR), y	
Group^b^	All	F	M	F	M
Positive	859	330	529	28.0 (24.0–36.0)	30.0 (25.0–39.0)
Negative	47_ _048	13_ _763	33_ _285	32.0 (26.0–44.0)	32.0 (24.0–44.0)
Inconclusive	16	6	10	-	-
Total	47_ _923	14_ _099	33_ _824	-	-
Positivity rate (95% CI)^c^	1.8 (1.7–1.9)	2.3 (2.1–2.6)	1.6 (1.4–1.7)		

Abbreviations: IQR, interquartile range; SARS-CoV-2, severe acute respiratory syndrome coronavirus 2.

^a^N = 47_ _923 unique participants tested 1–9 times from a total of 115_ _422 specimens with a positive, negative, or inconclusive result.

^b^Positive participants had at least 1 positive SARS-CoV-2 test result. Negative participants were defined as those who never had a positive result. Inconclusive patients had only an inconclusive test result, as defined in the “Methods.”

^c^The positivity rate was higher for women (Pearson chi-square *P* < .0001) and the median age for women testing positive (28) was lower than for positive men (30; Kruskal-Wallis *P* = .023).

**Table 3.** **Distribution of N1 and N3 Ct Values for Unobserved Self-Collected and Health Care Provider–Collected SARS-CoV-2-Positive Specimens**

Group	Indicated Symptomatic^a^	Period	Target	No.^b^	Ct, Median (IQR)	≥Shifted Ct, No. (%)^c^
SC	NA	6.3.20–8.12.20	N1	1268	32.7 (25.1–35.7)	67 (5.3)
			N3	1268	31.3 (24.0–34.2)	1 (0.08)
HCP	NA	6.3.20–8.12.20	N1	28_ _039	23.6 (17.4–30.8)	293 (1.0)
			N3	28_ _039	22.5 (16.6–29.5)	19 (0.07)
HCP	Yes	3.24.20–5.13.20	N1	4137	23.9 (17.5–33.0)	261 (6.3)
			N3	2753	22.9 (16.8–31.4)	4 (0.15)
HCP	No	3.24.20–5.13.20	N1	421	30.6 (22.0–36.4)	39 (9.3)
			N3	357	29.8 (22.2–34.9)	0 (0.0)

Abbreviations: Ct, cycle threshold; HCP, health care provider; IQR, interquartile range; RT-PCR, reverse transcription polymerase chain reaction; SARS-CoV-2, severe acute respiratory syndrome coronavirus 2; SC, self-collected.

^a^Client-reported prompted analyte that was optionally entered at the time of SARS-CoV-2 RT-PCR test order submission. Its use was discontinued in May 2020.

^b^Number of results available for the N1 and N3 targets. Before April 2020, the N1 and N2, but not the N3, targets were used in determining the test result. The age range for HCP-collected patients was set at 18–78 to match the age range for SC patients.

^c^The number of specimens with a Ct value greater than or equal to the pool-shifted Ct values of 37.6 (N1) and 38.1 (N3), as described in the “Methods” section.

Ct values for HCP-collected clinical specimens tested during the same time period were considerably lower than those for the SC cohort, with medians of 23.6 for N1 and 22.5 for N3 (Table 3). We also compared the Ct values from the SC and HCP-collected specimens with Ct values from patients who were indicated to be symptomatic or asymptomatic for COVID-19 on the test requisition; these specimens were tested during an earlier time period, when this indication was optionally provided at the time of test order placement. The median N1 and N3 Ct values for the specimens from the asymptomatic patients were not significantly different (N1: *P* = .29; N3: *P* = .39) than those from the SC cohort (Table 3). In contrast, the median N1 and N3 Ct values from the symptomatic patients were significantly lower (*P* < .0001) than those for the SC group and similar to those for the contemporarily HCP-collected clinical specimens (Table 3).

Previously, we described a SARS-CoV-2 pooled testing strategy to test 4 specimens in a single pool to increase testing efficiency and conserve testing resources [5]. When a single SARS-CoV-2-positive sample is pooled with 3 negative samples, there is a small loss of detection sensitivity, manifested as average Ct value shifts of 2.4 and 1.9 for the N1 and N3 targets, respectively, and the upper Ct cutoff for a positive result is then defined as 40 – Ctshift. To assess whether pooled testing could also be effective for unsupervised self-collected specimens without appreciable loss of sensitivity, we evaluated the effect of the pooled Ct value shift on the positive specimens in the current study. We found that 67 specimens (5.3% of the positives) had an N1 target Ct value greater than the pool-shifted Ct of 37.6 for N1 (Table 3). However, a single specimen had an N3 target Ct value greater than the pool-shifted Ct value of 38.1 for N3, and the N1 Ct for this specimen was 39.2. Thus, had pooled testing been utilized for these 1268 positive specimens, it is estimated that it would have resulted in only a single false-negative report and 66 inconclusive results, which would have necessitated repeat singlicate testing from the original specimen. We found similarly low proportions (0.0%–0.15%) of Ct values for HCP-collected clinical specimens that had N3 Ct values greater than the pool-shifted Ct value (Table 3).

## DISCUSSION

In this retrospective study of >47_ _000 individuals undertaking SARS-CoV-2 screening, unobserved self-collection of nasal swabs resulted in highly reproducible RP control Ct values with low interpatient variability. RP Ct values were <30 cycles for the vast majority of patients, indicating that unobserved self-collection is able to provide adequate swab samples for RT-PCR testing. The distribution of RP Ct values also did not vary between individuals with positive, negative, or inconclusive SARS-CoV-2 results. These findings alleviate concerns of increased false negatives in the context of unobserved self-collection for SARS-CoV-2 screening. Similar results have been found in other self-collection studies. In a study by Akmatov et al., the detection of a repsiratory viral pathogen was found to be independent of the DNA concentration of a β-actin internal adequacy control for both self-collected and staff-collected swabs [6]. The overall positivity rate for this screening population was low (<2%), and a higher percentage of women than men had positive test results. In contrast, the positivity rate for clinical HCP-ordered tests tested over the same period in our laboratory was nearly the same for men and women (9.7% and 9.9%, respectively). The reason for this sex difference in positivity rates in the self-collection cohort could not be ascertained; however, it could be speculated that women in this cohort assumed a larger proportion of tasks such as grocery shopping and child care that put them at higher risk for exposure to COVID-19.

Overall, 27% (N3 target) to 41% (N1 target) of the self-collected specimens had Ct values ≥33, representing lower viral loads in the specimens. For the 25% of patients with positive results who were serially tested in the self-collection cohort, Ct values from the last RT-PCR test obtained a median of 11 days after the first test had median Ct values of 33–34, a median of 9 Cts higher than the values obtained for the first positive test result (Figure 1). This increase in Ct values in serially tested specimens likely represents a decline in viral shedding [9]. Likewise, higher Ct values were seen for a set of HCP-ordered clinical specimens for patients indicated to be asymptomatic at the time of testing. In contrast, the median N1 and N3 Ct values from the symptomatic patients were significantly lower than those for the self-collection cohort and more similar to the contemporary HCP-collected clinical specimens (Table 3). We found that Ct values were significantly higher in the self-collected and asymptomatic cohorts. In prior work, asymptomatic patients have been shown to have viral loads comparable to those of symptomatic patients [10–13]. However, in 1 study of SARS-CoV-2 viral loads in asymptomatic and symptomatic children in pediatric hospitals, it was found that the adjusted median Cts for asymptomatic children were 10.3 Cts higher than for symptomatic children and the viral load was 3–4 logs lower [14]. The authors noted that asymptomatic patients with a recent known COVID-19 contact were more likely to have a higher viral load and postulated that the lower viral loads in the preprocedure/pre-admission testing groups likely reflected remote infections. In contrast, many of the studies of viral load level differences between asymptomatic and symptomatic adults involved well-defined cohorts with recent exposure [14]. Although data are not available for the timing of exposure for the cohorts in our study, it is plausible that differential exposure timing could account for these differences. Symptomatic patients are more likely to present during the window when the viral load is high. As noted among the serial-tested self-collected specimens, the Ct difference between the first positive and last positive test was a median of 9 Cts (Figure 1). In light of our findings, it is critical to understand the impact of the diluted pooled testing on the ability to provide accurate results when applying a pooled testing approach among asymptomatic patients.

In previous work [5], we showed that pooling 1 positive and 3 negative HCP-collected clinical specimens from populations with a SARS-CoV-2 prevalence of ≤10% does not increase the false-negative rate for RT-PCR testing. In the current study, we compared self-collected specimen Ct values with pool-shifted Ct values. Despite the higher median Ct values for this cohort, only a single specimen out of 1268 self-collected specimens could potentially have yielded a false-negative result in a pooled testing approach. An additional 67 specimens (5.3%) would have produced an inconclusive result, which would have then triggered single testing of the pooled specimens, resulting in 268 single tests. We again found that the low false-negative rate could be attributed to the use of a dual-target RT-PCR system that included the more sensitive N3 target. As the SARS-CoV-2 positivity rate for self-collected screening samples in return-to-work programs was low (1.8%), pooling could be utilized efficiently to reduce utilization of testing supplies and reagents while increasing laboratory testing throughput. Further gains in testing efficiency could be achieved by increasing the pool size from 4 specimens to 8, as recommended for maximizing pooled testing efficiency at this low prevalence (https://www.fda.gov/regulatory-information/search-fda-guidance-documents/policy-coronavirus-disease-2019-tests-during-public-health-emergency-revised). The effects of a larger pool size on assay sensitivity and the false-negative rate would need to be carefully assessed.

Our study had several limitations. First, for the majority of patients, no clinical information was available. Although most patients who are participating in a return-to-work SARS-CoV-2 testing program are likely to be asymptomatic, we cannot verify this claim. However, several lines of evidence suggest that most employees providing self-collected specimens were asymptomatic: (1) median Ct values were significantly higher in the self-collected specimens than in the HCP-collected clinical specimens tested over the same period, suggesting that they were drawn from different populations; (2) the median N1 and N3 Ct values in the self-collection cohort were significantly higher than the respective Ct values among patients indicated to be symptomatic at the time of testing; and (3) the median N1 and N3 Ct values in the self-collection cohort did not differ significantly from the respective Ct values for patients indicated to be asymptomatic at the time of testing.

Second, because this study was retrospective, we were unable to fully evaluate the performance of these specimens in pooled testing, as they were no longer available. Instead, we were able to estimate the performance of these specimens in pooled testing based on the reported single-specimen testing Ct values compared with the pool-shifted Ct values determined previously [5]. In-house validation studies have demonstrated reproducible Ct shifts with a 4-specimen Dorfman pooling scheme (data not shown). A prospective study would be needed to validate pooling for this population or to validate the use of a larger pool size to maximize testing efficiency.

Third, an RP internal adequacy control was not used for HCP-collected specimens, and thus we were unable to utilize RP Cts to evaluate potential differences between HCP collection and self-collection. As a control, we randomly tested specimens that had been HCP collected and found that they demonstrated similar RP Ct values to self-collected specimens (data not shown). Based on the literature, Akmatov et al. found that the median β-actin adequacy control DNA concentration was higher in self-collected vs staff-collected nasal swabs for respiratory pathogen detection [6]. Thompson et al. found that the mean RP Ct for self-collected nasal swabs used for influenza detection in pregnant women was significantly higher than the mean Ct for staff-collected nasopharyngeal swabs in an earlier study; however, the magnitude of the difference was modest (26.5 vs 24.1), and all specimens were adequate [8]. Arnold et al. compared the adequacy of participant-collected midturbinate or anterior nasal swabs vs staff-collected oropharyngeal swabs and again found that participant-collected swabs had modestly higher Cts, but the overall adequacy for participant-collected nasal specimens was 96.4% [7]. These findings alleviate the concern that the self-collected specimens in the current study had a higher SARS-CoV-2 false-negative rate than HCP-collected specimens.

Fourth, the specimen types for the HCP-collected specimens included a mix of specimen types and transport media, including nasal, nasopharyngeal, and oropharyngeal swabs, as well as other specimens. That information was not collected for the HCP specimens during the study period; therefore, we were unable to control for these factors when comparing the median Cts in HCP-collected specimens vs the self-collected nasal swab specimens. However, a recent review and meta-analysis found that the positive percent agreement in detection rates between nasal swab samples and a variety of index samples was high across a range of studies [15]. Tu et al. found that there was a good correlation between Cts for patient-collected nasal swabs and provider-collected NP swabs and noted that the Cts for the nasal swabs were lower than the Cts for the NP swabs in 50% of the cases and suggested that the viral loads in the nose and the nasopharynx may be equivalent [1].

Fifth, the cohort in this study consists primarily of working-age adults employed by major corporations, with >80% employed in the technology sector. The literacy rate may be higher in this group compared with the general population, reflecting on their ability to follow directions for self-collection and packaging and shipping of their specimens. For example, a study of 135 patients who self-collected swab samples for respiratory virus surveillance found that 13% of the participants made 1 or more packing errors when shipping the specimens [10]. Therefore, the high degree of adequate sampling we reported for the present cohort may not be generalizable to other diverse cohorts. However, our results were consistent with the patient usability study that was required for the EUA of an unobserved self-collection kit, which included a diverse cohort of patients (https://www.fda.gov/media/136231/download).

Lastly, the SARS-CoV-2 RT-PCR assay utilized in this study is a qualitative assay and not a quantitative assay for assessing viral load. Caution should be used when evaluating Ct values [9, 16]. While Ct values have been examined in a semiquantitative manner, it remains to be determined how Ct values correlate with transmission. Cell culture studies have shown that infectious virus is rarely isolated from individuals with viral loads <10^5^ copies/mL [11, 17]. It is unclear if these patients no longer harbored infectious virus at the viral loads represented by these Ct values, potentially representing a low transmission risk. Further studies are warranted to understand the dynamics of asymptomatic transmission. For a summary of duration and infectivity studies, see Rhee et al. [9].

In summary, we have demonstrated that unobserved self-collection of nasal swabs for SARS-CoV-2 does not increase the risk of false-negative results. This approach may be a good strategy for conserving PPE and minimizing risks of transmission to HCPs, patients, and screening participants. We further demonstrated that a more efficient pooled testing strategy could be used for this population as the likelihood of false-negative results is very low due to the use of a sensitive, dual-target RT-PCR test.

## Supplementary Data

Supplementary materials are available at Open Forum Infectious Diseases online. Consisting of data provided by the authors to benefit the reader, the posted materials are not copyedited and are the sole responsibility of the authors, so questions or comments should be addressed to the corresponding author.

ofab039_suppl_Supplementary_Materials
