# Supplementary material for: Performance of Unobserved Self-Collected Nasal Swabs for Detection of SARS-CoV-2 by RT-PCR Utilizing a Remote Specimen Collection Strategy
Source: Open Forum Infect Dis. 2021 Jan 28;8(4):ofab039. doi: 10.1093/ofid/ofab039 (PMC7928651; doi:10.1093/ofid/ofab039)
Supplement: ofab039_suppl_Supplementary_Materials [file ofab039_suppl_supplementary_materials.docx]

**A**


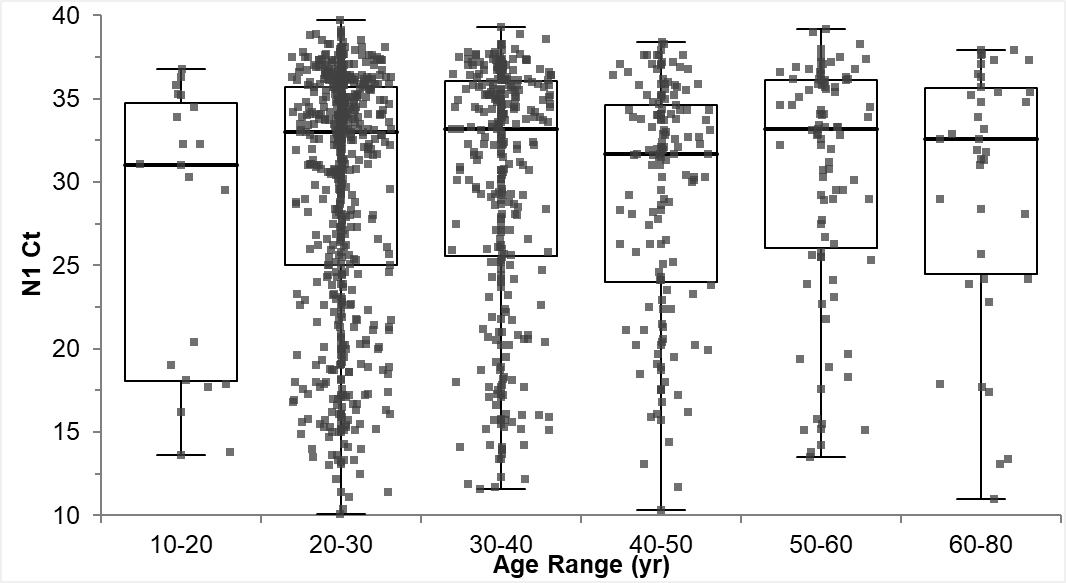


**B.**


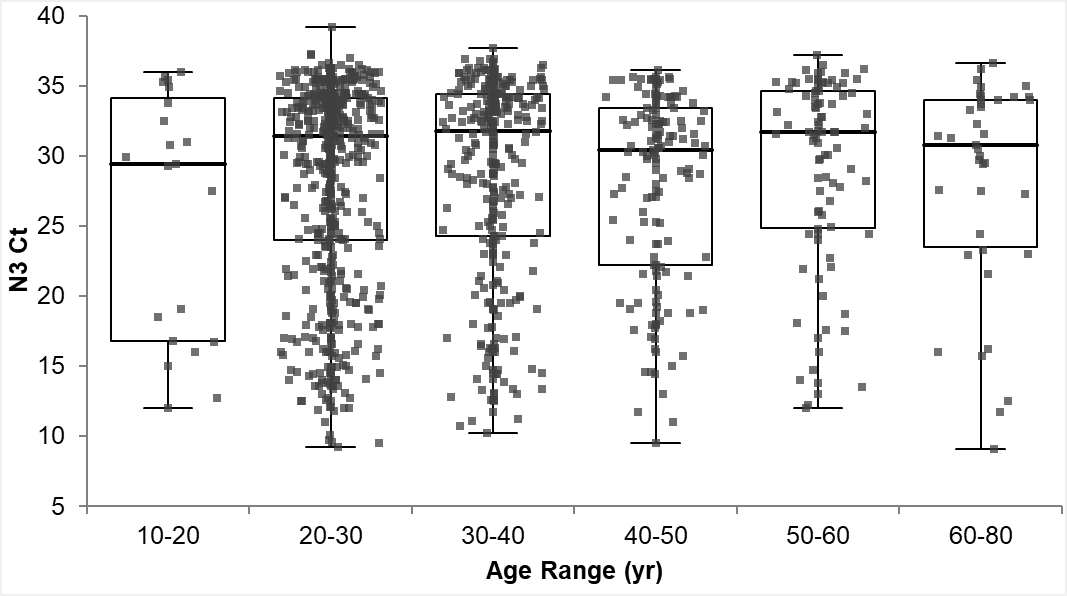


**Figure S1: Distribution of A. N1 Ct values and B. N3 Ct Values for positive patients stratified by age range. The differences in distributions were not statistically significant (Kruskal-Wallis test).**

**A**


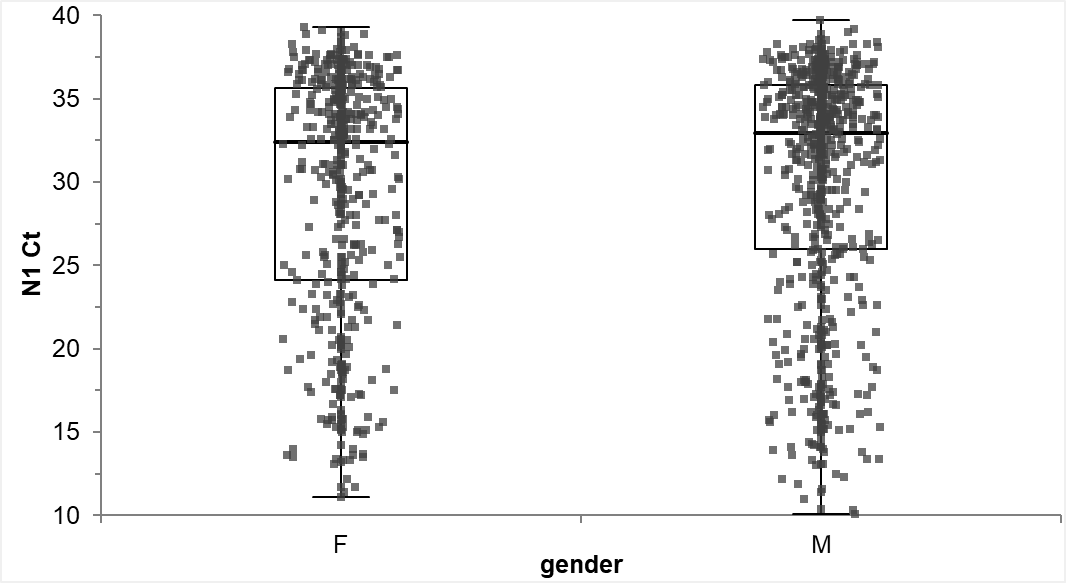


**B**


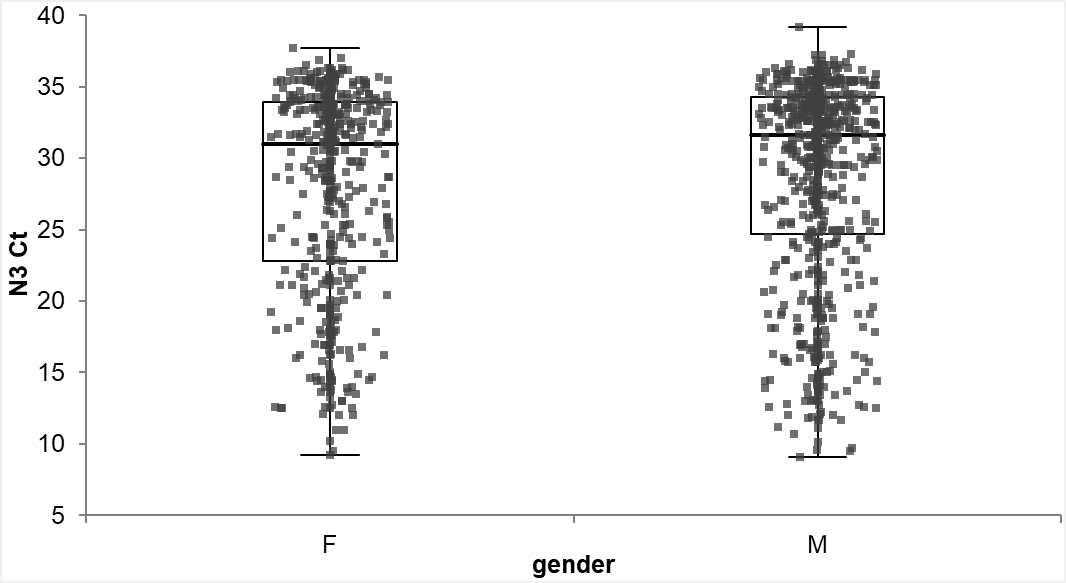


**Figure S2: Distribution of A. N1 Ct values and B. N3 Ct values for positive patients stratified by gender. The differences in distributions were not statistically significant (Kruskal-Wallis test).**


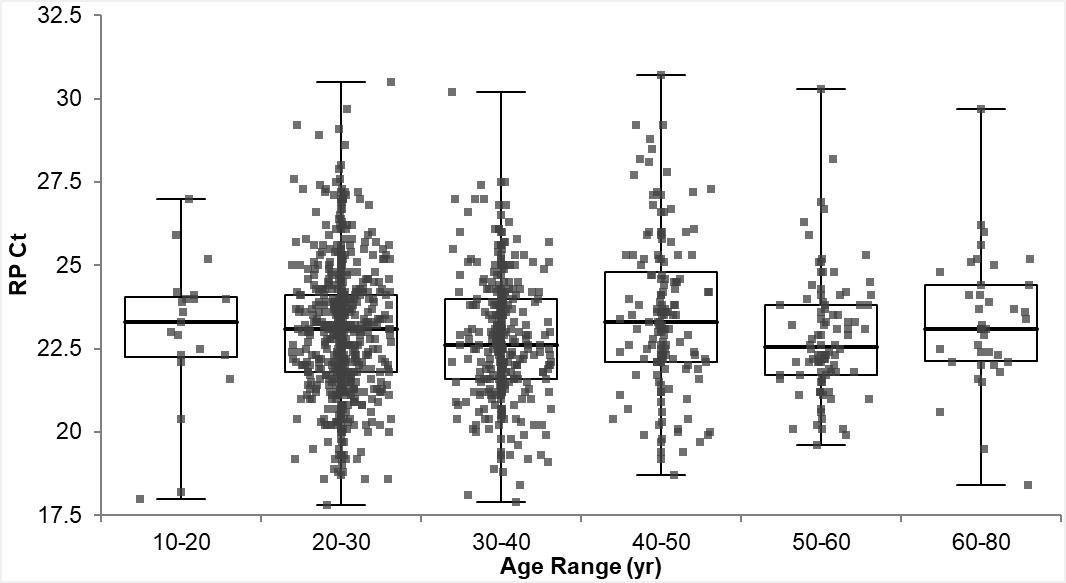


**Figure S3: Distribution of Rnase P Ct values for positive patients stratified by age range. The median Ct values ranged from 22.6 (30-40 yr and 50-60 yr age groups) to 23.3 (10-20 yr and 40-50 yr age groups) (p=0.016).**


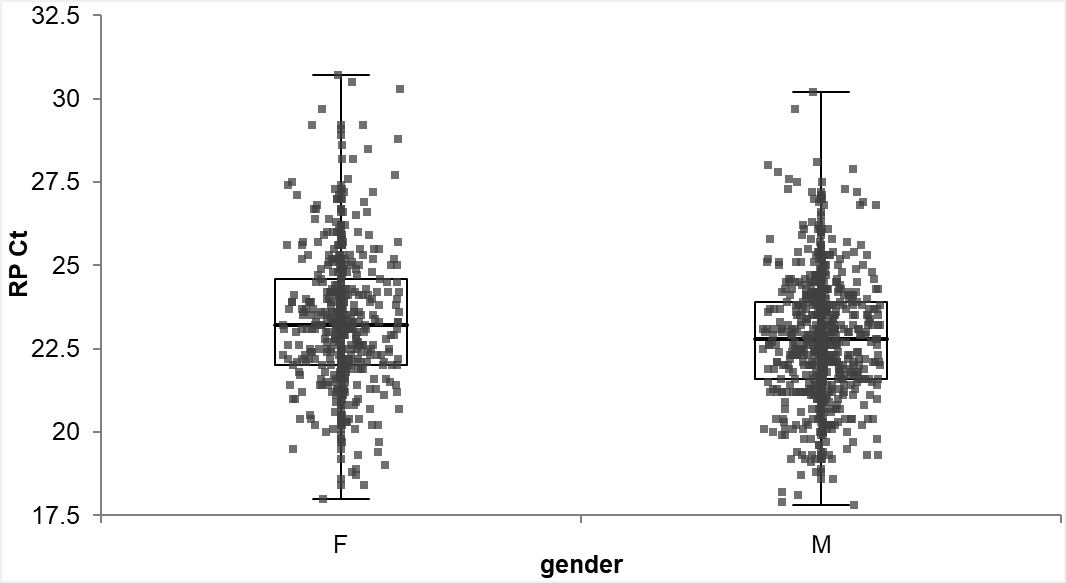


**Figure S4: Distribution of RP Ct values for positive patients stratified by gender. The medians for women (F) and for men (M) were 23.2 and 22.8 respectively, with a Hodges-Lehmann shift of 0.5 (95%CI: 0.3-0.7) Cts (p<0.0001)**
